# Sfrp1 protects against acute myocardial ischemia (AMI) injury in aged mice by inhibiting the Wnt/β-catenin signaling pathway

**DOI:** 10.1186/s13019-020-01389-4

**Published:** 2021-01-19

**Authors:** Jing Tao, Xian Wei, Ying Huang, Fen Liu, Yun Wu, Dilare Adi, Yang Xiang, You Chen, Yi-tong Ma, Bang-dang Chen

**Affiliations:** 1 Department of Cardiology, People’s Hospital of Xinjiang Uygur Autonomous Region, Urumqi, 830001 China; 2 Xinjiang Key Laboratory of Cardiovascular Disease, Clinical Medical Research Institute, First Affiliated Hospital of Xinjiang Medical University, Urumqi, Xinjiang 830054 People’s Republic of China; 3 Department of Cardiology, the First Affiliated Hospital of Xinjiang Medical University, Urumqi, 830054 China; 4 Department of Physiology, School of Preclinical Medicine, Xinjiang Medical University, Urumqi, Xinjiang 830011 People’s Republic of China

**Keywords:** Soluble frizzled related protein 1 (Sfrp1), Acute myocardial ischemia injury, Wnt/β-catenin pathway, Myocardial fibrosis, Aging

## Abstract

**Background:**

Aged patients suffering from acute myocardial ischemia (AMI) exhibit an increased mortality rate and worse prognosis, and a more effective treatment is currently in need. In the present study, we investigated potent targets related to Wnt/β-catenin pathway deregulation for AMI injury treatment.

**Methods:**

In the present study, AAV-Sfrp1 was transduced into the myocardium of aged mice, and an AMI model was established in these aged mice to study the effect and molecular mechanism of Sfrp1 overexpression on AMI-induced injury.

**Results:**

The results showed that Sfrp1 was successfully overexpressed in the myocardium of aged mice and remarkably reduced Wnt/β-catenin pathway activity in aged mice after AMI, effectively reducing the degree of myocardial fibrosis, inhibiting cardiomyocyte apoptosis, and improving cardiac function. We revealed that the exogenous introduction of Sfrp1 could be considered a promising strategy for improving post-AMI injury in aged mice by inhibiting Wnt/β-catenin pathway activity.

**Conclusions:**

In conclusion, the Wnt/β-catenin pathway potentially represents a key target in AMI in aged mice. Sfrp1 might be used as a small molecule gene therapy drug to improve heart function, reduce the degree of myocardial fibrosis, inhibit cardiomyocyte apoptosis and reduce AMI injury in aged mice by inhibiting the Wnt/β-catenin pathway, thereby effectively protecting aged hearts from AMI injury.

## Introduction

Cardiovascular disease (CVD) is a common disease in the elderly. The prevalence of atherosclerotic coronary artery disease (CAD) increases with increasing age, affecting more than half of those aged 60 years or older [1]. Therefore, in addition to the increased prevalence of acute myocardial ischemia (AMI), elderly patients are also associated with increasing levels of illness and death and worse prognosis, and a more effective treatment for AMI is currently in need [2]. Therefore, seeking new treatment measures to alleviate AMI injury in the elderly and improve the prognosis of elderly patients with AMI is an urgent problem that needs to be solved.

The Wnt/β-catenin signaling pathway is also termed the canonical Wnt signaling pathway, which participates in cell growth, development, proliferation, apoptosis and other important processes. Previous studies found that the Wnt/β-catenin pathway can be reactivated after MI and contributes to the modulation of pathophysiological processes, including ventricular remodeling, cardiomyocyte apoptosis, and myocardial fibrosis [3–5]. Under normal conditions, a balance exists between canonical and noncanonical Wnt signaling pathways within the myocardium, affecting the capacity of cardiac progenitor cells to proliferate and differentiate, respectively [6]. After cardiac injuries, myocardial cell apoptosis and fibrosis result in myocardial remodeling and cardiac failure [7]. Therefore, effectively inhibiting the Wnt/β-catenin pathway may have important therapeutic value within the prevention and treatment of AMI injury in elderly patients [8, 9].

Soluble frizzled-related protein 1 (Sfrp1), which shares a similar cysteine-rich domain with the frizzled receptor in the Wnt/β-catenin pathway, competitively targets the interstitially circulating Wnt ligand, thereby blocking the transduction of the Wnt/β-catenin pathway into cells while negatively regulating the Wnt/β-catenin pathway [10]. Our previous study found that Sfrp1 overexpression can effectively reduce Wnt/β-catenin signaling pathway activity and reduce the mortality of chronic cardiac failure within aged mice [11]. However, the possibility that Sfrp1-mediated inhibition of Wnt/β-catenin signaling activity effectively inhibits MI injury in aged mice is worthy of further study. At present, in the cardiovascular field, the effects of Sfrp1 in the prevention and treatment of AMI injury in elderly people and the underlying molecular mechanism have not been thoroughly investigated.

Therefore, in the present study, we generated Sfrp1 overexpression in aged mice by tail vein injection of AAV-Sfrp1 and established an AMI model in these mice. Cardiomyocyte morphological changes and cardiac function were evaluated; fibrosis progression and cardiac extracellular matrix (ECM) deposition were examined. In addition, cardiomyocyte apoptosis was assessed, and alterations within the protein levels of key Wnt/β-catenin signaling factors were monitored. In summary, we attempt to provide a solid experimental basis for understanding the role and mechanism of Sfrp1 overexpression in improving post-AMI injury via the Wnt/β-catenin pathway.

## Materials and methods

### Establishment of an AMI injury model in mice

Male C57BL/6 J mice aged 15 months (weighing 28 ~ 35 g) were obtained from the Experimental Animal Center of Xinjiang Medical University. All animal experiments were performed following a protocol approved by the Institutional Animal Care and Use Committee at the First Affiliated Hospital of Xinjiang Medical University. The experimental mice were housed in an SPF environment (12-h:12-h day and night cycle) with free access to food and water. Two weeks later, after the mice were acclimated to the new environment, the mice were randomly assigned to five experimental groups.

**Fig. 1:**
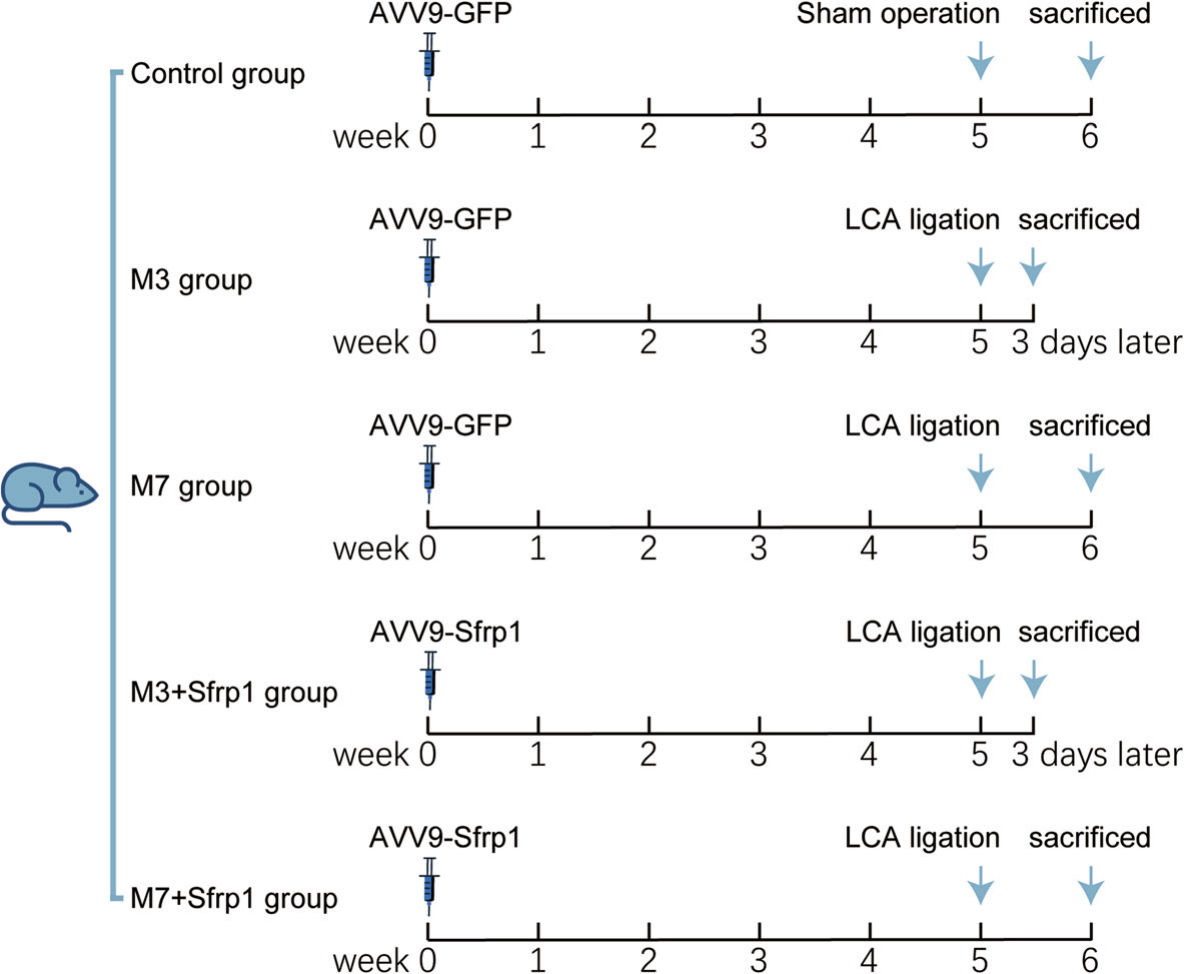
The experiments programs on aged mice. Mice were divided into 5 groups. Control group: mice received tail vein injections of dsAVV9-GFP for 5 weeks, and then the chest of the mice was open without left coronary artery (LCA) ligation; M3 group: mice received tail vein injections of dsAVV9-GFP for 5 weeks and then subjected to the LCA ligation and sacrificed 3 days later; M3 + Sfrp1 group: mice received tail vein injections of dsAVV9-Sfrp1 for 5 weeks and then subjected to the LCA ligation and were sacrificed 3 days later; M7 group: mice received tail vein injections of dsAVV9-GFP for 5 weeks and then received the LCA ligation and were sacrificed 7 days later; M7+ Sfrp1 group: mice received tail vein injections of dsAVV9-Sfrp1 for 5 weeks and then subjected to the LCA ligation and were sacrificed 7 days later

Mice were assigned to the following groups. In the control group (sham operation group, *n* = 15), mice received tail vein injections of dsAVV9-GFP (1 × 10^11^ vg/mice) for 5 weeks. Then, the chest was opened, but the left coronary artery (LCA) was not ligated. In the M3 group (*n* = 28), mice received tail vein injections of dsAVV9-GFP for 5 weeks and were subsequently subjected to LCA ligation following the methods described previously [12–14]. Mice in this group were sacrificed 3 days after the LCA ligation. In the M3 + Sfrp1 group (*n* = 30), mice received tail vein injections of dsAVV9-Sfrp1 (1 × 10^11^ vg/mice) for 5 weeks, were subjected to LCA ligation and were sacrificed 3 days later. In the M7 group (*n* = 27), mice received tail vein injections of dsAVV9-GFP for 5 weeks, underwent LCA ligation and were sacrificed 7 days later. In the M7 + Sfrp1 group (*n* = 28), mice received tail vein injections of dsAVV9-Sfrp1 for 5 weeks and were then subjected to the LCA ligation and sacrificed 7 days later. The experimental procedures are shown in Fig. 1. Successful modeling was characterized as follows: under the surgical microscope, the left coronary artery innervation area was paler and had segmental dysplasia compared with the surrounding myocardial tissue; the electrocardiogram suggested ST-segment elevation or QRS wave widening malformation and persisted.

### Echocardiography

Echocardiography analysis was performed using a Sonos 5500 ultrasound system (Hewlett-Packard, Palo Alto, CA, USA). Mice were anesthetized by injection of compound anesthetic containing 0.1 g of ketamine, 1 mg of atropine, and 5 mg of xylazine in 10 ml of physiological saline (0.1 ml/10 g weight). Mice could spontaneously breathe and were placed on a table with a thermostatic pad. Then, posterior wall thickness in diastole (Pwdth), left ventricular end-diastolic dimension (LVEDd), and left ventricular short-axis shortening rate (FS) were measured. All measurements were measured by double-blind methods by two physicians.

### Histological analysis

Hematoxylin-eosin (HE) staining and Masson’s staining was applied to visualize cardiomyocyte morphological changes and cardiac fibrosis area according to previous description [15, 16].

### Immunoblotting

Proteins extracted from tissues or cells were separated by SDS-PAGE, transferred to a polyvinylidene difluoride membrane (Millipore, Burlington, MA, USA), and then incubated with the primary antibody followed by appropriate secondary antibody (Abcam, Cambridge, MA, USA). The following primary antibodies were used: anti-TrkB (ab18987), anti-Akt (ab32505), anti-p-Akt (ab81283), anti-Erk (ab54230), anti-p-Erk (ab50011), anti-ADRB2 (ab182136), anti-PKA (ADI-KAS-PK017-F; Enzo, Hong Kong, China), anti-p-PKA (ab75991), and anti-caveolin-3 (ab2912). All antibodies were obtained from Abcam unless otherwise stated.

### TUNEL staining

Cardiomyocyte apoptosis was examined using a TUNEL kit (Beijing Zhongshan Biotechnology Co.) following the instructions. Under a 200× light microscope, five visual fields were randomly selected and counted to calculate the apoptosis index of cardiomyocytes. Cardiomyocyte apoptosis index = number of apoptotic cells/total number of cardiomyocytes × 100%.

### Immunohistochemical (IHC) assay

IHC was performed to detect the locations and protein levels of Col-1 according to previous description [15]. Optical microscopy was performed to visualize section images. ImageJ software was used to analyze the data.

### Statistical analysis

Data are expressed as the mean ± SD. Statistical analysis was performed using one-way analysis of variance (ANOVA)) following Tukey’s tests (GraphPad Prism 6) were applied for multiple comparisons between groups. *P* < 0.05 was considered statistically significant.

## Results

### In vivo effects of Sfrp1 on histology and cardiac function of aged mice with AMI

**Fig. 2:**
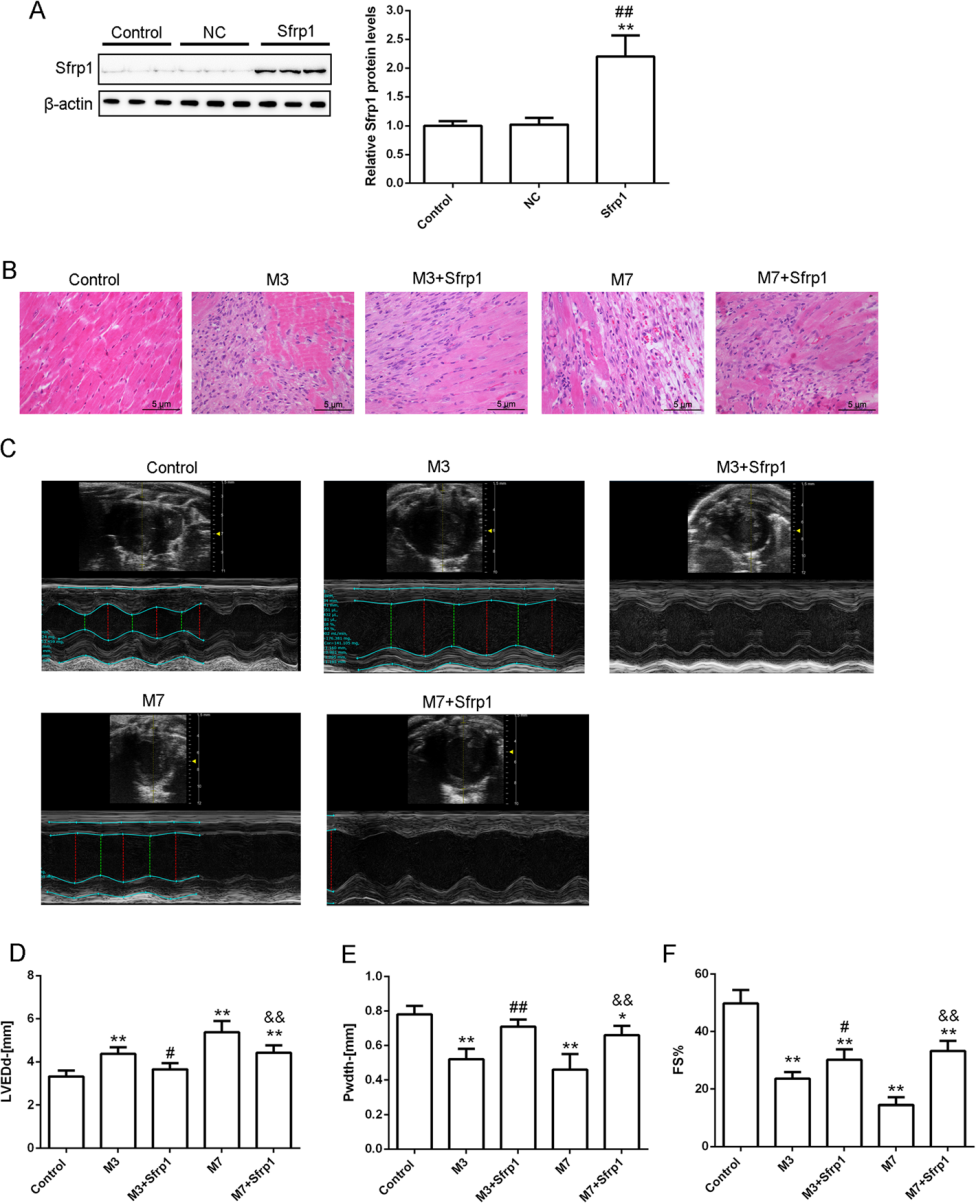
In vivo effects of Sfrp1 on histology and cardiac function of aged mice with acute myocardial infarction (AMI) (**a**) The infection efficiency of AAV-Sfrp1 was examined by immunoblotting. **b** Cardiac histopathological changes evaluated by HE staining. **c**-**f** Cardiac function evaluated by echocardiography. *P < 0.05, **P < 0.01, compared to the control group; #*P* < 0.05, ##*P* < 0.01, compared to the NC group or M3 group; &&*P* < 0.01, compared to the M7 group

AAV-Sfrp1 was injected to generate the overexpression of Sfrp1; the infection efficiency of AAV-Sfrp1 was examined 5 weeks after the first injection by immunoblotting (Fig. 2a). HE staining results (Fig. 2b) demonstrated that the myocardial fibers within the control group were neatly arranged, and the cardiomyocyte texture was clear. After 3 days of MI, some inflammatory cells infiltrated the infarcted area. In addition, the cardiomyocyte membrane was incomplete, and the cardiomyocytes were disordered. After 7 days of MI, cardiomyocyte necrosis, myocardial fiber rupture, myocardial fiber lysis vacuolization, myocardial fiber gap widening, and fibrous tissue in the infarct area were observed. After Sfrp1 overexpression, inflammatory cell infiltration was decreased compared to the M3 group; myocardial necrosis and fibroplasia symptoms were dramatically decreased compared to the M7 group.

Representative ultrasound images of each group exhibited typical changes in cardiac structure after MI (Fig. 2c). After MI, the left ventricular diameter gradually increased, and the left ventricular wall motion gradually decreased. Sfrp1 overexpression improved MI-induced changes within the M3 + Sfrp1 group and the M7 + Sfrp1 group. Specifically, LVEDd increased, Pwdth became thinner, and the FS decreased significantly in the M3 + Sfrp1 group compared to the control group (Fig. 2d-f and Table 1). The cardiac function parameters of the M7 group deteriorated further (Fig. 2d-f and Table 1). Compared with the MI group (M3 and M7 groups), after overexpression of Sfrp1, the enlarged ventricle was partially improved, manifesting as decreased LVEDd and relatively increased Pwdth and FS (Fig. 2d-f and Table 1). This finding suggested that Sfrp1 could inhibit myocardial remodeling and improve cardiac function.

**Table 1:** In vivo effects of Sfrp1 on cardiac function of aged mice with acute MI

	Control	M3	M3 + Sfrp1	M7	M7 + Sfrp1
LVEDd (mm)	3.32 ± 0.28	4.37 ± 0.31^**^	3.65 ± 0.29^#^	5.37 ± 0.53^**^	4.42 ± 0.35^**&&^
Pwdth (mm)	0.78 ± 0.05	0.52 ± 0.06^**^	0.71 ± 0.04^##^	0.46 ± 0.09^**^	0.66 ± 0.05^*&&^
FS (%)	49.84 ± 4.63	23.56 ± 2.32^**^	30.12 ± 3.67^**#^	14.45 ± 2.64^**^	33.26 ± 3.49^**&&^

*P < 0.05, **P < 0.01, compared to control group; #*P* < 0.05, ##*P* < 0.01, compared to NC group or M3 group; &&*P* < 0.01, compared to M7 group

### In vivo effects of Sfrp1 on cardiac ECM deposition

**Fig. 3:**
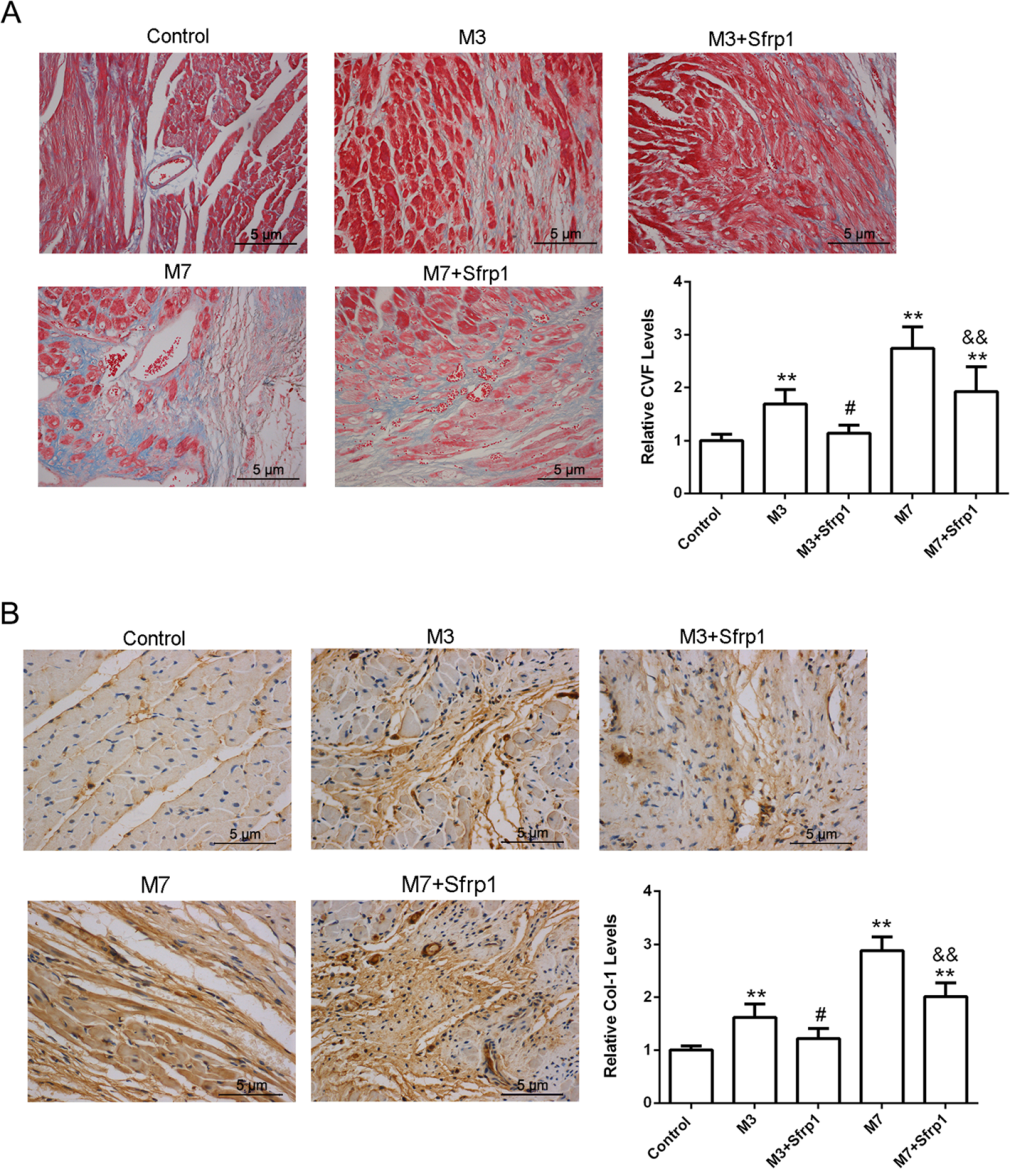
In vivo effects of Sfrp1 on cardiac extracellular matrix (ECM) deposition. **a** Collagen volume fraction (CVF) evaluated by Masson’s staining in each group. **b** Col-1 localization and protein levels were evaluated by IHC staining in each group. **P < 0.01, compared to the control group; #*P* < 0.05, compared to the M3 group; &&*P* < 0.01, compared to the M7 group

Next, cardiac fibrous hyperplasia was evaluated by Masson’s staining and IHC staining. As shown in Fig. 3a and Table 2, the collagen volume fraction (CVF) was dramatically enhanced within the M3 group and M7 group and increased more in the M7 group, suggesting that the collagen fibers were hyperplastic and that the organization structure in the MI area was damaged. After Sfrp1 overexpression, the collagen fibers were significantly reduced in both the M3 + Sfrp1 and M7 + Sfrp1 groups (Fig. 3a and Table 2), indicating that Sfrp1 could effectively inhibit cardiac fibrous hyperplasia and improve MI injury.

**Table 2:** In vivo effects of Sfrp1 on CVF, Col-1 levels and apoptosis rate

	Control	M3	M3 + Sfrp1	M7	M7 + Sfrp1
CVF levels	1.00 ± 0.12	1.69 ± 0.27^**^	1.14 ± 0.15^#^	2.74 ± 0.41^**^	1.92 ± 0.47^**&&^
Col-1 levels	1.00 ± 0.08	1.62 ± 0.25^**^	1.22 ± 0.19^#^	2.88 ± 0.26^**^	2.01 ± 0.26^**&&^
Apoptosis rate (%)	9.23 ± 1.72	32.15 ± 3.43^**^	21.76 ± 2.22^**##^	47.33 ± 5.12^**^	23.34 ± 2.86^**&&^

**P < 0.01, compared to control group; #*P* < 0.05, compared to M3 group; &&*P* < 0.01, compared to M7 group

Col-1 in the cardiomyocyte extracellular matrix (ECM) is a crucial component of the cardiac interstitium. Here, IHC staining (Fig. 3b and Table 2) results showed that Col-1 was only moderately expressed in the myocardial interfiber space in the control group. After 3 days of MI, the arrangement of cardiomyocytes was disordered and Col-1 expression increased. After 7 days of MI, the myocardial fiber structure was destroyed, the myocardial fiber gap was widened, and Col-1 expression was further increased sharply. After Sfrp1 overexpression, Col-1 expression was significantly downregulated compared to the corresponding MI group, suggesting that abnormal deposition of myocardial ECM (Col-1) is improved by Sfrp1 overexpression and that myocardial fibrosis is reduced.

### In vivo effects of Sfrp1 on cardiomyocyte apoptosis

**Fig. 4:**
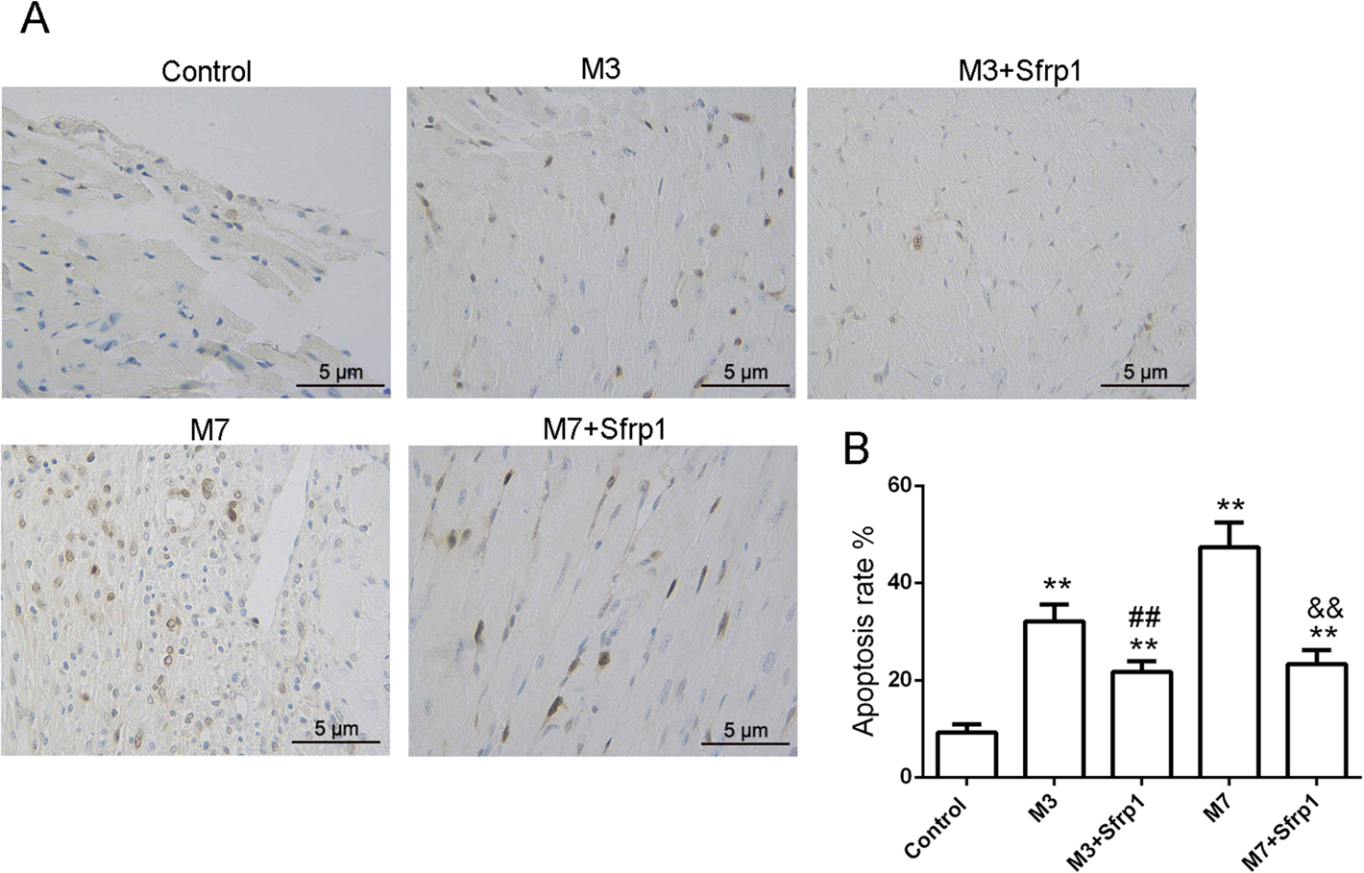
In vivo effects of Sfrp1 on cardiomyocyte apoptosis Cardiomyocyte apoptosis was evaluated by TUNEL staining in each group. **P < 0.01, compared to the control group; ##*P* < 0.01, compared to the M3 group; &&*P* < 0.01, compared to the M7 group

Cardiomyocyte apoptosis was evaluated by TUNEL staining in each group. The results showed that the cardiac myocyte apoptosis rate was significantly enhanced after MI in a time-dependent manner (Fig. 4 a-b). However, when Sfrp1 was overexpressed, the cardiaomyocyte apoptosis rate was obviously reduced in the M3 + Sfrp1 group and M7 + Sfrp1 group compared to the corresponding MI group (Fig. 4 a-b and Table 2), indicating that Sfrp1 overexpression can effectively inhibit cardiomyocyte apoptosis in aged mice with AMI injury.

### In vivo effects of Sfrp1 on the Wnt/β-catenin signaling pathway in aged mice with acute MI

**Fig. 5:**
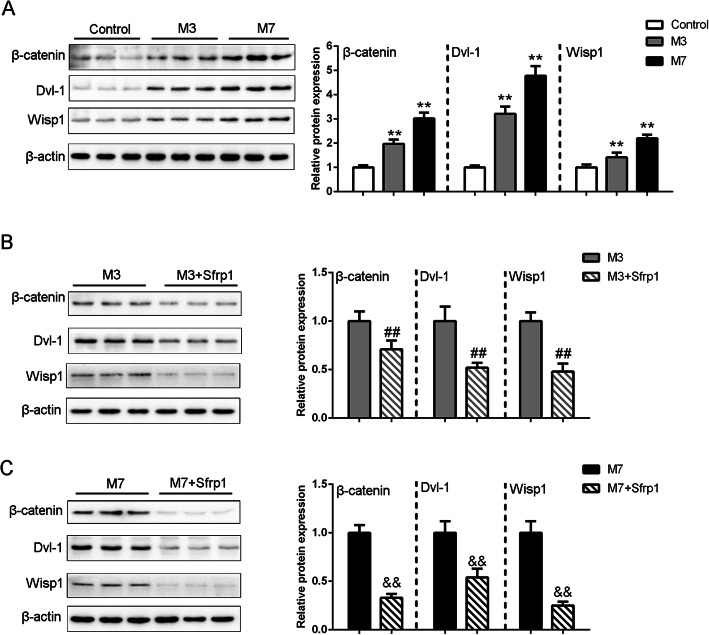
In vivo effects of Sfrp1 on Wnt/β-catenin signaling in aged mice with AMI (**a-c**) β-catenin, Dvl-1, and Wisp1 protein levels were determined by immunoblotting in each group. **P < 0.01, compared to control group; ##*P* < 0.01, compared to M3 group; &&*P* < 0.01, compared to M7 group

We further investigated the effects of Sfrp1 overexpression on key factors in the Wnt/β-catenin signaling pathway. β-catenin, Dvl-1, and Wisp1 protein levels were determined by immunoblotting. MI significantly enhanced β-catenin, Dvl-1, and Wisp1 protein levels in a time-dependent manner (Fig. 5a and Table 3). After Sfrp1 overexpression, MI-induced increases in β-catenin, Dvl-1, and Wisp1 protein levels were significantly reduced in both the M3 + Sfrp1 and M7 + Sfrp1 groups (Fig. 5b-c and Table 4). In summary, the Wnt/β-catenin pathway can be continuously activated within 3 to 7 days after MI; however, Sfrp1 overexpression significantly inhibits Wnt/β-catenin pathway activation induced by MI.

**Table 3:** The relative expression of β-catenin, Dvl-1 and Wisp1 in control, MI groups

	Control	M3	M7
β-catenin	1.00 ± 0.09	1.97 ± 0.18^**^	3.02 ± 0.24^**^
Dvl-1	1.00 ± 0.08	3.21 ± 0.3^**^	4.78 ± 0.4^**^
Wisp1	1.00 ± 0.12	1.42 ± 0.19^**^	2.2 ± 0.15^**^

**P < 0.01, compared to control group

**Table 4:** In vivo effects of Sfrp1 on the relative expression of β-catenin, Dvl-1 and Wisp1 protein levels

	M3	M3 + Sfrp1	M7	M7 + Sfrp1
β-catenin	1.00 ± 0.10	0.71 ± 0.09^##^	1.00 ± 0.08	0.33 ± 0.04^&&^
Dvl-1	1.00 ± 0.15	0.52 ± 0.05^##^	1.00 ± 0.12	0.54 ± 0.09^&&^
Wisp1	1.00 ± 0.09	0.48 ± 0.08^##^	1.00 ± 0.12	0.25 ± 0.04^&&^

##*P* < 0.01, compared to M3 group; &&*P* < 0.01, compared to M7 group

## Discussion

Herein, AAV-Sfrp1 carrying the Sfrp1 gene was transduced into the myocardium of aged mice, and an AMI model was established in these aged mice to study the effect and molecular mechanism of Sfrp1 overexpression on AMI-induced injury in aged mice. The results showed that after transduction of AAV-Sfrp1, Sfrp1 was successfully overexpressed in the myocardium of aged mice and could remarkably reduce Wnt/β-catenin pathway activity in aged mice after AMI, effectively reducing the degree of myocardial fibrosis, inhibiting cardiomyocyte apoptosis, and improving cardiac function. We revealed that exogenous introduction of Sfrp1 could be considered a promising strategy for improving post-AMI injury in aged mice by inhibiting Wnt/β-catenin pathway activity.

Sfrp1 inhibits the Wnt/β-catenin signaling pathway [17–19]. There are 5 types Sfrps, and Sfrp1 is not only present throughout heart development and adulthood but is also expressed substantively within both the mouse heart and human heart [20]. Sfrp1 plays a critical role in the proliferation of vascular cells in vitro and in vivo [21]. In addition, Sfrp1 can help improve the structure and function of the heart after myocardial infarction within rodents [22]. Our previous study also showed that Sfrp1 prevents the H9C2 cell injury caused by hypoxia/reoxygenation in vitro [23]. However, these findings are not based on aged animal models or senescent cell models. In the present study, Sfrp1 overexpression in aged mice partially inhibited myocardial remodeling, improved cardiac function, reduced inflammatory cell infiltration, improved myocardial necrosis and fibroplasia symptoms, inhibited cardiac fibroplasia, improved the abnormal deposition of cardiac ECM (Col-1), decreased the degree of myocardial fibrosis, and inhibited the apoptosis rate of cardiomyocytes. In general, Sfrp1 overexpression can effectively reduce the degree of myocardial fibrosis in the early stage of AMI, inhibit cardiomyocyte apoptosis, and thus improve post-AMI injury healing and cardiac function in aged mice.

The Wnt pathway exerts an important effect on CVD pathogenesis [24]. The Wnt pathway can be reactivated postinjury and during multiple pathological states or reparation processes [25]. Baurand et al. [26–28] found that β-catenin deletion and mutation inhibited the Wnt/β-catenin signaling pathway, reduced left ventricular remodeling, and improved heart function. β-catenin overexpression reversed Wnt/β-catenin pathway activation and resulted in progressive development of dilated cardiomyopathy [28]. Zelarayán et al. [27] found that compared with wild-type mice, conditional β-catenin-knockout mice exhibited a significant decrease in MI area after 4 weeks post-MI, and the survival rate of MI mice was effectively improved. Studies have also shown that Dvl-1 knockdown can block the Wnt/β-catenin signaling pathway, and stress-induced cardiac hypertrophy is improved [29]. Herein, β-catenin, Dvl-1, and Wisp1 protein levels were significantly increased after AMI in a time-dependent manner. However, after Sfrp1 overexpression, these proteins were significantly reduced, indicating that Sfrp1 might exert its beneficial effects on AMI injury by inhibiting the Wnt/β-catenin signaling pathway.

Regarding the limitations of the present study, many studies have confirmed the protective effect of Sfrp1 inhibiting Wnt/β-catenin signaling pathway activity in the AMI model of young mice; thus, in the present study, we investigated the role of Sfrp1 inhibiting Wnt/β-catenin signaling pathway activity in aged mice. However, to compare the indicated process in young and aged mice in future study would help to fully understand the characteristics of Sfrp1 inhibiting Wnt/β-catenin signaling pathway activity. Considering the lower housing cost compared to large animals, shorter gestation times, reduced costs for pharmacological treatments, and easier delivery of AAVs that target the myocardium of small rodents [30], we chose aged mice as the experimental animal models in the present study. However, large animals [31, 32], such as pig, dog, sheep, and non-human primates, should be taken into consideration in future study. Although the present study provided solid experimental basis (in vitro in cell model and in vivo in mice model) for understanding the role of Sfrp1 inhibiting Wnt/β-catenin signaling pathway activity in acute myocardial infarction, there are more investigations should be finished before clinical trial. Firstly, use large animal models, such as dog, pig, sheep, and non-human primates, which could better mimic the anatomical characteristics and physiological function as those of human. Secondly, monitor the systematic changes in related factors in animal models to investigate whether the administration of AAVs targeting myocardium might cause systematic toxicity. Thirdly, developing efficient targeted-delivery manner could reduce potential off-target and subsequent systematic toxicity.

## Conclusion

**Fig. 6:**
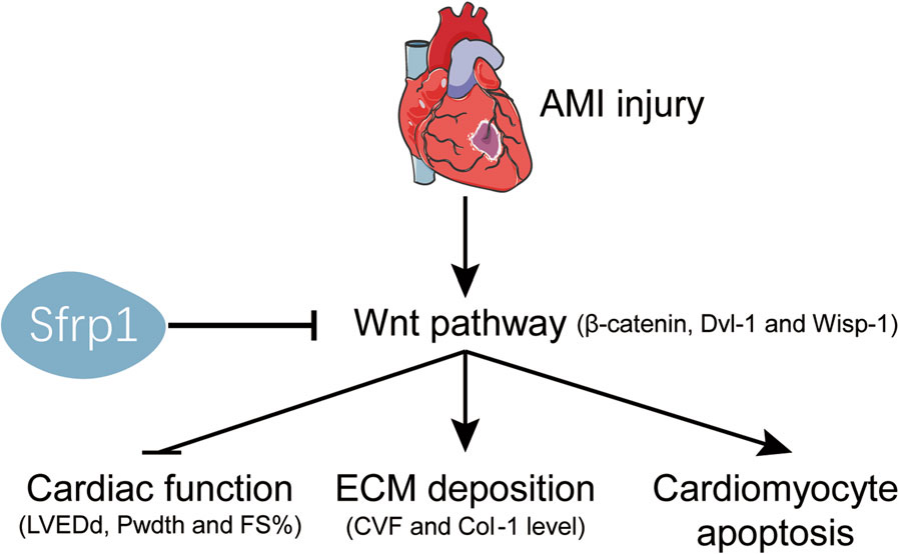
A possible mechanism of Sfrp1-attenuated AMI injury in aged mice. Sfrp1 improved cardiac function, reduced ECM deposition and cardiomyocyte apoptosis in AMI injured aged mice by inhibiting Wnt/β-catenin pathway. → indicates promotion or activation; ┴ indicates inhibition or reduction

The Wnt/β-catenin pathway represents a key target in AMI in aged mice; early after AMI, the Wnt/β-catenin pathway was shown to be continuously activated. Sfrp1 might be used as a small molecule gene therapy drug to improve heart function, reduce the degree of myocardial fibrosis, inhibit cardiomyocyte apoptosis and reduce AMI injury in aged mice by inhibiting the Wnt/β-catenin pathway, thereby effectively protecting aged hearts from AMI injury (Fig. 6).

## Data Availability

All available data were present in this study.

## Abbreviations

AMI: Acute myocardial ischemia
Sfrp1: Soluble frizzled related protein 1
CVD: Cardiovascular disease
CAD: Coronary artery disease
ECM: Extracellular matrix
LCA: Left coronary artery
Pwdth: Posterior wall thickness in diastole
LVEDd: Left ventricular end-diastolic dimension
HE: Hematoxylin–eosin
IHC: Immunohistochemical
DAB: Diaminobenzidine
CVF: Collagen volume fraction
